# Trends in epidemics pertaining to notifiable infectious diseases in China and prediction models for key diseases: a case study of Ziyang County

**DOI:** 10.1186/s12889-025-25482-2

**Published:** 2025-11-21

**Authors:** Siqi Xu, Lin Chen, Jian Luo, Bosen Hu

**Affiliations:** 1 Ziyang County Center for Disease Control and Prevention, Ankang, Shaan Xi 725300 China; 2 Shenzhen Center for Chronic Disease Control, Shenzhen, 518020 China; 3 Dapuqiao Community Health Center, Huangpu District, Shanghai, 200023 China; 4 Department of Preventive Medicine and Health Care, Dapuqiao Community Health Center, No. 712 Liyuan Road, Huangpu District, Shanghai, 200023 China

**Keywords:** Infectious disease trends, SARIMA model, Epidemiology, Epidemic forecasting

## Abstract

**Background:**

Notifiable infectious diseases remain a public health priority in China. Understanding long-term epidemiological patterns and developing low-cost, automated forecasting tools may support timely prevention efforts at the county level.

**Methods:**

We analyzed 26,756 valid cases of 29 statutory infectious diseases reported in Ziyang County (2013–2023). Diseases with ≥ 500 cumulative cases were selected for primary analysis. Seasonal autoregressive integrated moving average (SARIMA) models were fitted for tuberculosis (TB), influenza, and hand–foot–mouth disease (HFMD), with two training windows (2013–2021 and 2018–2021) used for influenza to account for surveillance updates. Model selection was based on the Akaike information criterion, and forecasts were validated using data from 2022–2023.

**Results:**

TB, influenza, and HFMD accounted for more than 55% of the cases. The TB incidence steadily decreased, with spring–summer peaks, whereas the incidence of influenza increased after 2019 and peaked in the winter. Short-horizon forecasts were robust across training windows but less accurate in 2022–2023, likely reflecting post-COVID-19 behavioral and policy shifts. HFMD forecasts were unstable because of zero-inflated data.

**Conclusions:**

The incidence of tuberculosis decreased consistently, whereas the incidence of influenza rebounded sharply after nonpharmaceutical interventions were lifted. The SARIMA models reproduced seasonal patterns but showed limited accuracy for influenza and HFMD, reflecting the influence of surveillance changes, behavioral shifts, and sparse data. These findings highlight the need for complementary approaches—such as models explicitly accounting for structural breaks—to improve the reliability of local infectious disease forecasts.

**Supplementary Information:**

The online version contains supplementary material available at 10.1186/s12889-025-25482-2.

## Background

Research on the historical trends that characterize epidemics pertaining to notifiable infectious diseases is highly important with respect to efforts to develop and improve epidemic surveillance systems [1, 2]. A comprehensive description of past rates can provide a more accurate understanding of the historical development of infectious diseases in a given region, thus providing a scientific basis for predictions regarding future epidemics [3]. Moreover, county-level Centers for Disease Control and Prevention (CDCs) can analyze historical data with the aim of identifying local risk factors, transmission routes, and vulnerable populations, thus enabling them to implement timely and targeted preventive measures [4, 5]. Understanding past epidemic patterns is not only beneficial with respect to resource allocation and the formulation of vaccination strategies but can also ensure that the CDC can respond rapidly in the event of an outbreak. Time series models have been widely used to predict trends pertaining to infectious diseases, and the seasonal autoregressive integrated moving average (SARIMA) model is a common analytical tool in this context [4, 6, 7]. 

In many county-level CDCs in China, particularly in less developed regions, staffing and analytic resources remain constrained, with limited availability of personnel trained in advanced statistics or modeling [8, 9]. Against this backdrop, an important practical question is whether simple, low-cost, and automated forecasting tools can provide actionable insights for frontline disease control. The present study therefore analyzed the epidemic trends of notifiable infectious diseases in Ziyang County, Ankang City, Shaanxi Province, from 2013 to 2023 to provide an overview of the general levels and epidemiological characteristics of various diseases. Furthermore, the SARIMA model was applied to forecast short-term trends using historical data, with the dual aim of evaluating where such automated methods may be effective and where they encounter limitations, thereby delineating their real-world applicability in resource-limited public health settings.

## Methods

### Data sources

Case information for notifiable infectious diseases in Ziyang County (2013–2023) was obtained from individual case reports submitted through the Chinese CDC Infectious Disease Reporting System. The case date was defined as the date of diagnosis recorded in the system. Population denominators for 2013, 2018, and 2023 were obtained from statistical yearbooks. For intervening years, we estimated population sizes using two-point linear interpolation between adjacent benchmark (census) years, assuming a linear change as follows [10]. Let t₀ and t₁ denote the adjacent benchmark years for any year t with$\:{t}_{0}<t<{t}_{1},\:{P}_{t}={P}_{t0}+\frac{{P}_{t1}-{P}_{t0}}{{t}_{1}-{t}_{0}}\times\:\left(t-{t}_{0}\right)$.

As of 2023, the updated national list by the National Disease Control and Prevention Administration included 41 notifiable infectious diseases. We excluded any nonnotifiable conditions (e.g., pneumoconiosis) to restrict analyses to legally notifiable infectious diseases. For the primary disease analysis, we prespecified an inclusion threshold of > 500 cumulative cases during 2013–2023 to ensure statistical power.

Occupation- or identity-based population groups were defined according to national standards used in the reporting system. For subgroup analyses, we prespecified the inclusion of groups with ≥ 200 cumulative cases during 2013–2023 to ensure statistical robustness; cases coded as “unknown” were retained in overall counts but were excluded from subgroup analyses because of limited epidemiological interpretability.

## Descriptive study

A retrospective analysis was conducted with the aim of describing the epidemiological characteristics of major notifiable infectious diseases in Ziyang County from 2013 to 2023. Key indicators such as the number of cases, incidence rate, and composition ratio were used to analyze the temporal and demographic distributions of infectious diseases.

### Seasonal pattern summaries and concordance

To summarize the recurrent seasonality while aligning with the SARIMA training window, we computed month-of-year seasonal indices for 2013–2021 at both the county and population-group levels. For each year *y* and month *m*, we first calculated the within-year monthly proportion $\:{p}_{y,m}={C}_{y,m}/{\sum\:}_{k=1}^{12}{C}_{y,k}$, where $\:{C}_{y,m}$ denotes the monthly case count. Seasonal indices were then obtained by averaging these proportions across years, yielding a 12-month profile $\:{\left\{{I}_{m}\right\}}_{m=1}^{12}$ per stratum. Months with missing counts were treated as zero for that year; years with zero annual counts contributed no information (all proportions undefined) and were excluded from the cross-year average.

From each 12-month profile, we derived two descriptive quantities: the peak month (the month with the maximal seasonal index) and amplitude (peak-to-trough ratio, $\:max{I}_{m}/min{I}_{m}$; undefined if the minimum is zero). To gauge alignment between county-level and group-specific seasonality, we defined concordance within ± 1 month as the fraction of the six major groups (children, students, farmers, homemakers, retirees, and commercial services) whose peak month fell within ± 1 calendar month of the county peak (months treated cyclically). These measures are descriptive and noninferential; they are reported as counts and proportions (percentages). The resulting indices support the qualitative comparison of seasonal windows with county-level SARIMA patterns presented in the Results section.

### Time series model and forecasting

A time series analysis was performed via the “auto_arima” function, which was drawn from the pmdarima library on the Python 3.10 platform, to fit the optimal seasonal autoregressive integrated moving average (SARIMA) model automatically. The SARIMA model was chosen because it extends the classical autoregressive integrated moving average (ARIMA) framework by incorporating seasonal autoregressive and moving average terms, which is appropriate for modeling the well-established annual seasonality of infectious diseases.

SARIMA models were trained on data from January 2013 through December 2021 (January 2018 through December 2021 for influenza sensitivity analysis) and validated against observed data from January 2022 through December 2023 to enable a two-year out-of-sample performance assessment.

The auto_arima routine performs a stepwise search across a prespecified parameter grid to identify the model with the lowest Akaike information criterion (AIC). The search space was defined as follows:

- Nonseasonal autoregressive (AR) and moving average (MA) orders: 0–5
- Seasonal AR and MA orders: 0–2
- Differencing: up to 2 nonseasonal differences and 1 seasonal difference
- Seasonal period: 12 months

Candidate models were automatically generated and compared. The maximum number of model fitting iterations was 100, and the lowest-AIC model was selected per disease [11].

To ensure relevance and stability, forecasting was a priori restricted to three diseases that met prespecified criteria informed by the occupational-group composition rankings (Fig. 3) and prior evidence: (i) high county-level cumulative burden; (ii) coverage of distinct epidemiologic profiles across occupational groups (adult-focused, child/student-focused, and cross-cutting); and (iii) clear, recurrent monthly seasonality. Accordingly, tuberculosis (TB), hand–foot–mouth disease (HFMD), and influenza were selected as representative targets. For diseases with sparse or zero-inflated monthly counts—at or below the scale observed for HFMD—subgroup SARIMA models were not used because of expected instability; instead, group-specific seasonality was summarized descriptively to align with county-level forecasts.

## Sensitivity analysis of training windows for influenza forecasting

To evaluate the robustness of the influenza forecasts under surveillance-driven data shifts, we conducted a sensitivity analysis using two distinct training windows. During the study period, China’s influenza surveillance framework underwent important updates. In 2017, the National Influenza Surveillance Work Plan refined sentinel network operations and laboratory testing workflows, and subsequent editions of the Influenza Diagnosis and Treatment Guidelines (notably in 2020) expanded the use of rapid antigen and nucleic acid amplification tests (NAATs) as case confirmation criteria [12, 13]. These changes plausibly increased the sensitivity of case detection and reporting after 2017 and particularly after 2020, thereby introducing nonstationary shifts in the observed case counts independent of true transmission intensity.

Accordingly, we prespecified two training windows: 2013–2021 (long window) and 2018–2021 (post-2017 window). SARIMA models were trained separately on each window, and forecasts for 2022–2023 were compared to assess whether surveillance-driven discontinuities substantially altered model fit or predictive performance. This analysis was not intended to identify an “optimal” historical window but rather to quantify the sensitivity of the results to surveillance changes. Ideally, influenza forecasts would be normalized using denominators based on influenza-like illness (ILI) consultations at sentinel outpatient clinics, as recommended by the World Health Organization (WHO) and national surveillance guidelines [14]. However, county-level ILI denominators were not routinely collected or accessible during the study period; thus, we modeled absolute case counts and population-based incidence rates instead. The implications of this approach are further elaborated in the Limitations section.

### Statistical methods

Descriptive statistics and data transformations were performed using Microsoft Excel 365. Count data were summarized as rates or composition ratios. Group comparisons of composition ratios were conducted using the chi-square test. For pairwise comparisons among diseases, we performed two-sided tests on the two-category margin (binomial/2 × 2 chi-square) and controlled the familywise error rate using the Holm step-down procedure; Holm-adjusted p values are reported. The same procedure was applied within each major population group in the sensitivity analyses. Temporal trends in annual incidence were assessed using Spearman’s rank correlation between the calendar year and the corresponding measure. All tests were two-sided, with *α* = 0.05.

For population group rankings, the top five diseases within each major group were determined by cumulative case counts for 2013–2023 (rather than incidence rates), as prespecified.

Time series modeling and forecasting were performed with Python 3.10.8 using pmdarima 2.0.4. Forecast performance was assessed for the 2022–2023 holdout period using the mean absolute error (MAE), root mean squared error (RMSE), mean absolute percentage error (MAPE), coefficient of determination (R²), and 95% prediction interval (PI) coverage, computed for both multistep (static) forecasts and rolling one-step-ahead forecasts. PI coverage was defined as the percentage of observed monthly counts falling within the nominal 95% PIs.

## Results

### Overall incidence of major notifiable infectious diseases from 2013 to 2023

**Fig. 1:**
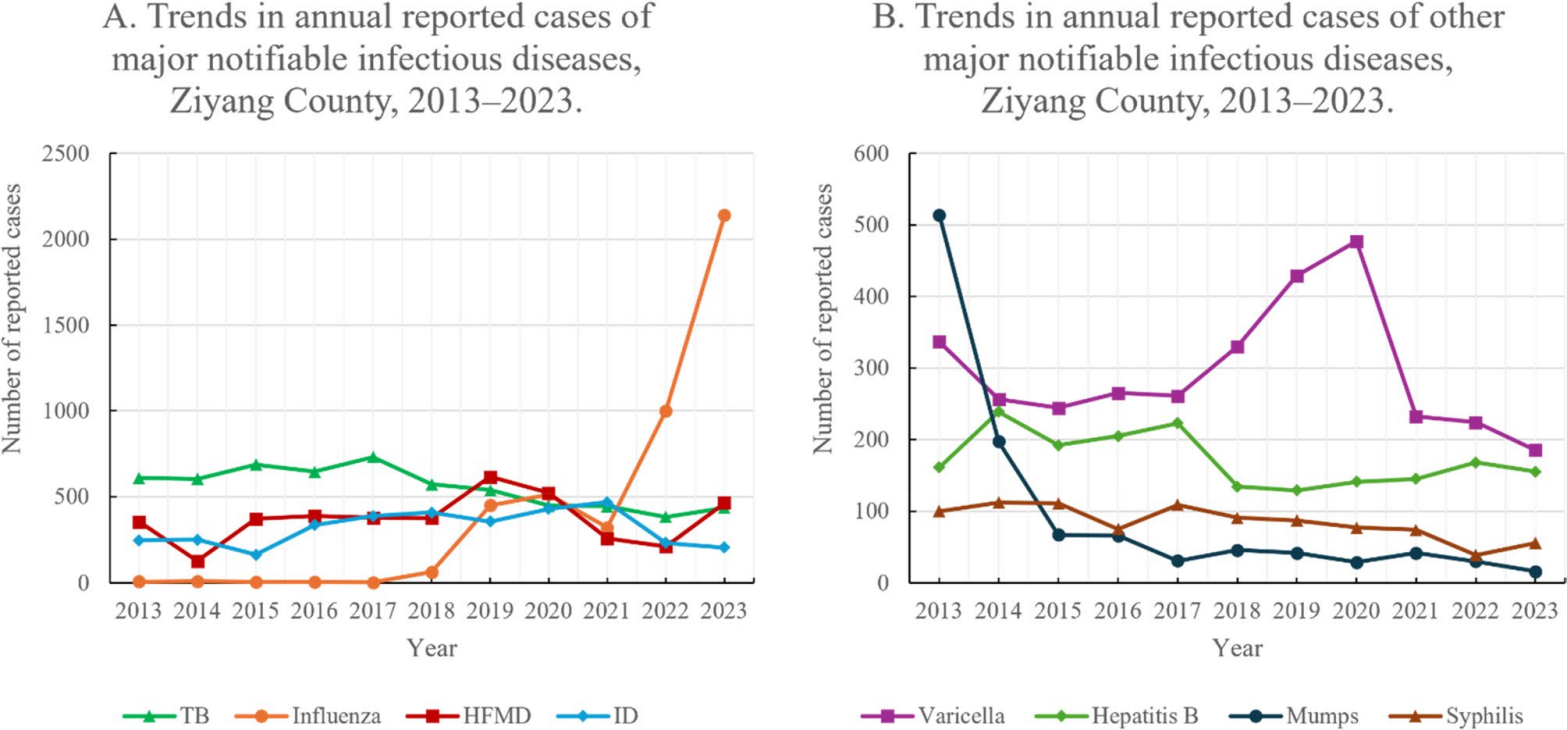
Trends in annual reported cases of the eight most common notifiable infectious diseases, Ziyang County, 2013–2023. **A** TB, influenza, HFMD, and ID. **B** Varicella, hepatitis B, mumps, and syphilis

Between 2013 and 2023, a total of 27,276 case reports were submitted to Ziyang County. After exclusions, 26,756 notifiable cases remained for analysis. Among the 29 notifiable diseases reported at least once during this period, eight met the prespecified inclusion threshold and were retained for the primary analysis. The annual trends of these eight diseases, grouped by disease type, are shown in Fig. 1. Eight diseases, TB, influenza, HFMD, other infectious diarrheal disease (ID), varicella, hepatitis B, mumps, and syphilis, accounted for more than 90% of all reported cases. Complete annual case counts and the incidence rates for all 29 locally reported notifiable diseases are provided in Supplementary Table S1 (Parts a–e). The proportions differed significantly across diseases $\:\left({\chi\:}^{2}\right[28]=\:84850.164,\:p\:<\:0.001$). Among the 406 pairwise contrasts, adjacent pairwise comparisons with Holm adjustment were significant among the upper-ranked diseases (e.g., TB–influenza, influenza–HFMD, and HFMD–ID), whereas ID and varicella had comparable proportions, closely following HFMD (Holm-adjusted *p* = 0.17; see Supplementary Table S1f).

TB had the highest cumulative and average annual incidence. Its incidence significantly decreased from 2013 to 2023 (*p* < 0.01). Influenza ranked second in terms of cumulative cases and average annual incidence; its incidence increased markedly after 2019, indicating a significant temporal change (*p* < 0.01). HFMD ranked third, followed by ID.

### Reported incidence rankings of notifiable infectious diseases among major populations from 2013 to 2023

The top five notifiable infectious diseases within each of the six major population groups in Ziyang County during 2013–2023 (ranked by cumulative case counts as prespecified in the Methods section) are shown in Fig. 3. Overall, the composition of reported diseases varied across population groups. Among children and students, hand–foot–mouth disease (HFMD) and varicella were the most commonly reported conditions. Within-group compositions differed significantly $$\begin{aligned} &(\text{c}\text{h}\text{i}\text{l}\text{d}\text{r}\text{e}\text{n}:\:{\chi\:}^2\lbrack17\rbrack=\:\text{40,222.25},\:p\:<\:0.001;\\ &\text {students} :{\chi\:}^2\lbrack17\rbrack=\:\text{18,801.44},\:p\:<\:0.001) \end{aligned}$$ , and in both groups, all the Holm-adjusted pairwise contrasts among the five highest-share diseases were significant (adjusted *p* < 0.05).

In contrast, tuberculosis (TB) ranked first among farmers, commercial service workers, and retirees and ranked second among homemakers. Influenza appeared among the top five diseases across all major population groups. Farmers: $\:\left({\chi\:}^{2}\right[25]=\:\text{78,142.16},\:p\:<\:0.001)$ All the top five pairwise contrasts were significant after the Holm adjustment (adjusted *p* < 0.001), except for hepatitis C vs. syphilis (adjusted *p* = 1.00). Commercial service workers: The within-group composition differed significantly$\:\left({\chi\:}^{2}\right[12]=\:224.67,\:p\:<\:0.001)$. However, adjacent pairwise contrasts in the rank order—including within the top five—were not statistically significant after the Holm adjustment (all adjusted *p* = 1.00); thus, neighboring ranks are described as “similar in share”. Retirees: $\:\left({\chi\:}^{2}\right[10]=\:484.61,\:p\:<\:0.001)$ Within the top five diseases, TB vs. each of the other four was significant (adjusted *p* < 0.001), while all other pairwise contrasts were not significant (all adjusted *p* = 1.00). Homemakers: The within-group composition differed significantly $\:\left({\chi\:}^{2}\right[17]=\:1539.75,\:p\:<\:0.001)$. However, adjacent pairwise contrasts among the five highest-share diseases were not statistically significant after the Holm adjustment (influenza–TB: *p* = 1.00; TB–syphilis: *p* = 0.13; syphilis–hepatitis B: *p* = 1.00; hepatitis B–varicella: *p* = 0.61).

**Fig. 2:**
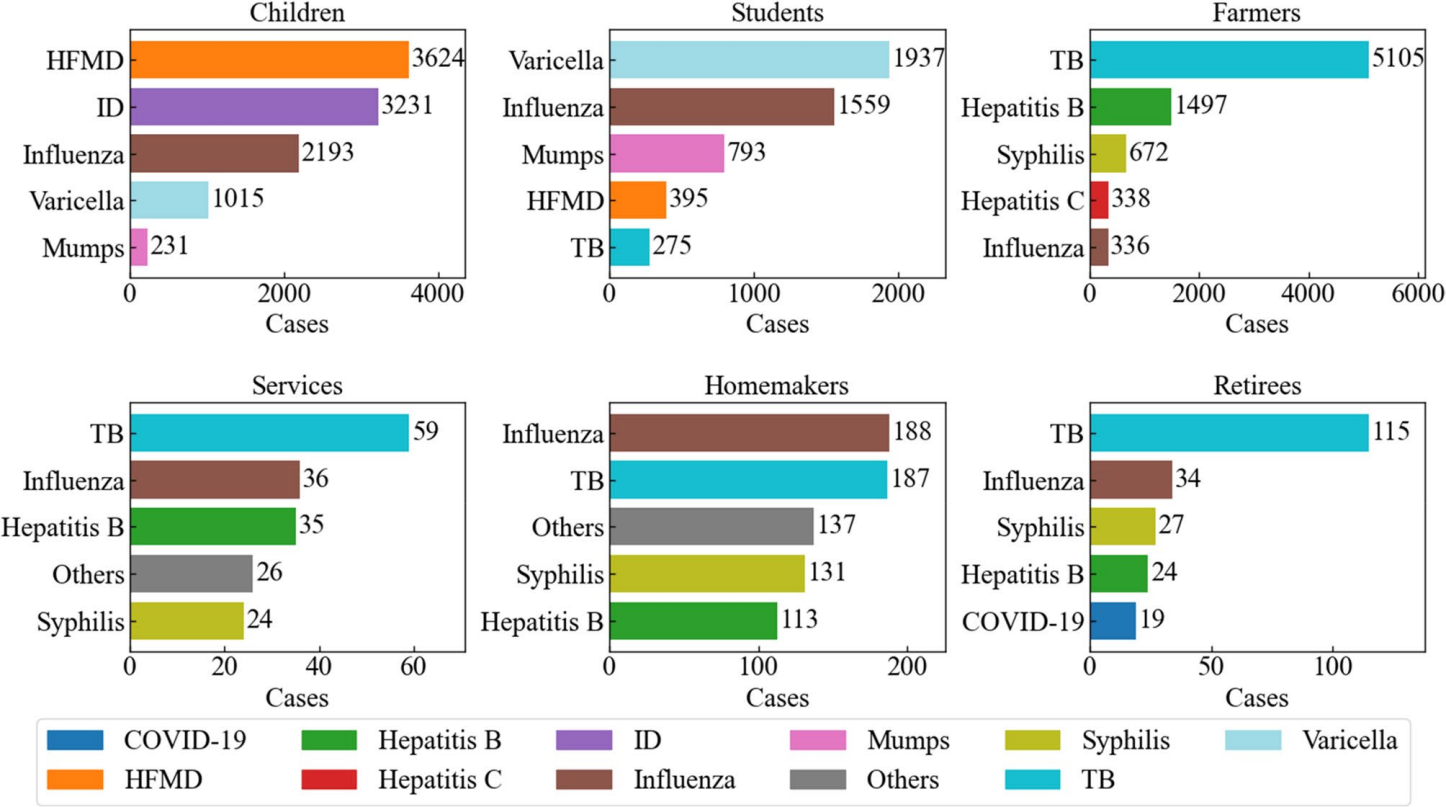
Top 5 notifiable infectious diseases by population group, Ziyang County (2013-2023)

The complete case counts for all 18 occupation/identity groups are summarized in Supplementary Table S2. Per the prespecified threshold, six major groups—together accounting for > 95% of all reported cases—were retained for the ranking analysis (Fig. 2). These within-group rankings also informed the forecasting scope: TB (adult-dominated), HFMD (child/student-dominated), and influenza (present across all groups) were carried forward to SARIMA modeling (see Methods). The remaining groups were not analyzed separately.

### Prediction of infectious disease trends on the basis of the SARIMA model

**Fig. 3:**
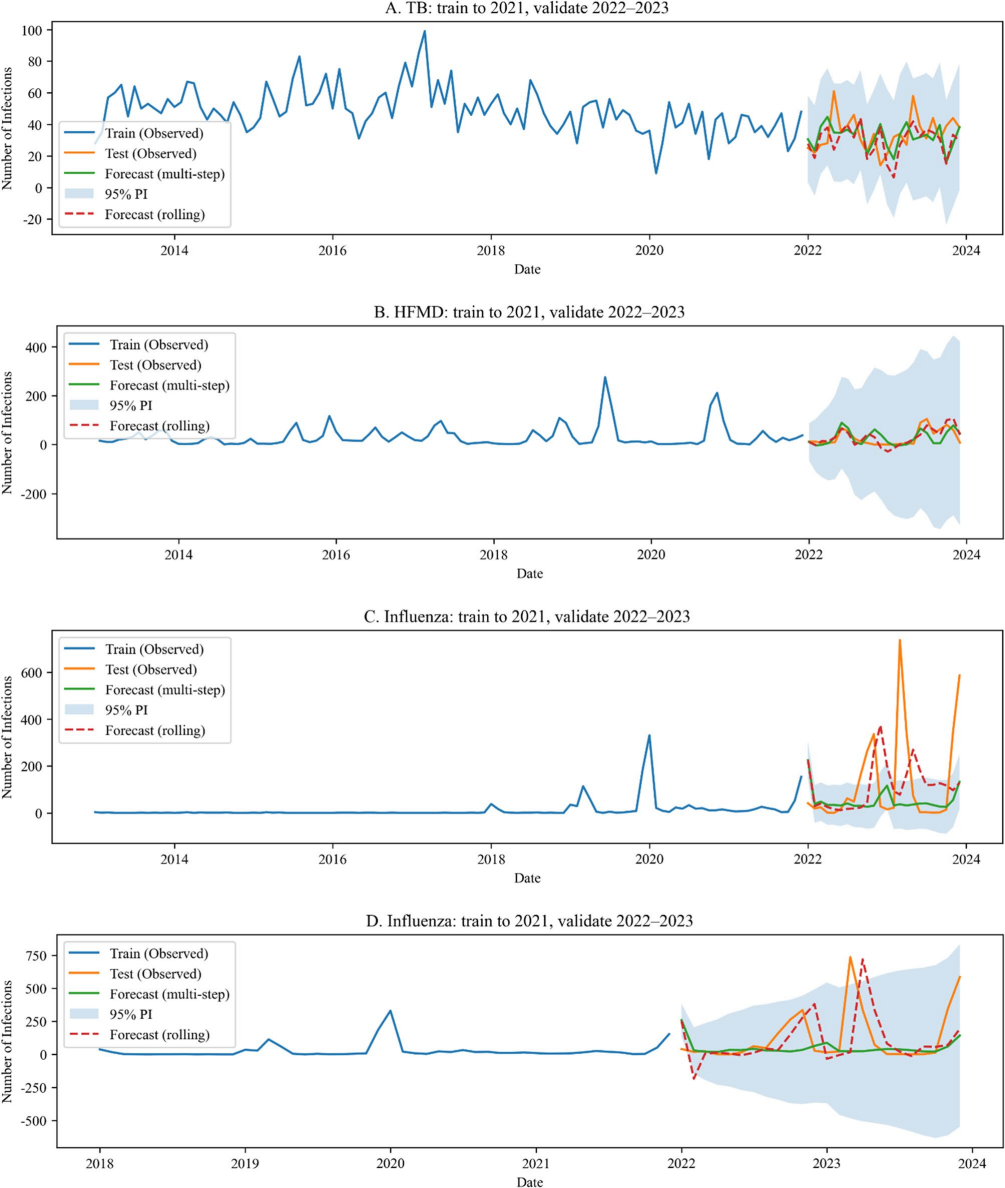
Prediction of Infectious Disease Trends on the Basis of the SARIMA Model. **A** TB; (**B**) HFMD; (**C**) Influenza (2013–2021); (**D**) Influenza (2018–2021)

Before forecasting, we summarized month-of-year seasonal indices (2013–2021), which showed county-level peaks in July for tuberculosis (TB), January for influenza, and June for hand–foot–mouth disease (HFMD), with peak-month concordance within ± 1 month across the six major population groups of 2/6 (TB), 4/6 (influenza), and 2/6 (HFMD) (Supplementary Fig. S1). These observed seasonal windows are broadly consistent in peak timing with those later recovered by the county-level SARIMA models (see Fig. 3), suggesting a two-tier operational approach in which county-level forecasts define preparedness windows, while group-specific profiles fine-tune the timing and targeting of interventions.

We developed seasonal ARIMA (SARIMA) models for TB, HFMD, and influenza to forecast the monthly incidence in Ziyang County. These three high-burden diseases together accounted for approximately 55% of all locally reported notifiable cases during 2013–2023 (Supplementary Table S1) and were therefore prioritized for forecasting. As prespecified in the Methods section, model selection used the minimum Akaike information criterion (AIC); the top-ranked candidate fits and AIC values are summarized in Supplementary Table S3, and the final selected specifications are shown in Table 1.

**Table 1:** Forecast evaluation metrics for three infectious diseases, Ziyang County, 2022–2023

Disease	MAE (M/*R*)	RMSE (M/*R*)	MAPE% (M/*R*)	*R*^2^ (M/*R*)	PI Cov. %	PI Width
TB	10.7/10.7	13.7/13.9	34.0/33.7	−0.58/−0.62	100	69.4
HFMD	22.6/18.7	28.7/23.1	321.0/251.4	0.21/0.49	100	514.5
Influenza (2013)	129.8/155.9	213.9/217.1	553.3/1533.7	−0.18/−0.21	63	196.3
Influenza (2018)	127.2/145.8	215.5/225.8	451.1/592.0	−0.19/−0.31	92	882.0

PI Cov. = prediction interval coverage at 95% confidence; PI width = average prediction interval width

*M* multistep forecast, *R* rolling forecast

The final models were SARIMA(2,1,1)(2,1,0)[12] for TB, SARIMA(0,1,0)(2,1,1)[12] for HFMD, and SARIMA(0,1,1)(1,1,1)[12] for influenza (2013–2021 training window). For influenza, a shorter training window (2018–2021) yielded a model of SARIMA(0,1,0)(1,1,0)[12]. The fitted values, multistep and rolling forecasts, and 95% prediction intervals are shown in Fig. 3.

TB forecasts suggested a continued decline with seasonal peaks in late spring/early summer. HFMD forecasts approached a near-zero baseline and included negative values in some months, reflecting instability under sparse counts. Influenza forecasts indicated year-on-year increases, with winter remaining the primary epidemic season. The forecast accuracy metrics (MAE, RMSE, MAPE, R², and 95% prediction interval coverage) are reported in Table 1.

Robustness (influenza). Use of the 2013–2021 training window vs. the 2018–2021 training window produced different information criteria but similar qualitative predictions for 2022–2023 (Table 1; Fig. 3C–D). The absolute forecast errors were large in 2022–2023.

The forecast performance was assessed using the MAE, RMSE, MAPE, R², and 95% prediction interval (PI) coverage, where the PI coverage is defined as the percentage of observed monthly counts falling within the nominal 95% prediction interval; metrics were computed for both multistep (static) and rolling one-step-ahead forecasts for 2022–2023.

## Discussion

In the present study, 26,756 valid cases of notifiable infectious diseases were reported across 18 occupation categories between 2013 and 2023. For analytical clarity, we focused on the six population groups with ≥ 200 reported cases—children, students, farmers, homemakers, retirees, and commercial service workers—together accounting for more than 96% of all the cases (Supplementary Table S2). Across these groups, TB, influenza, and HFMD remained the most prevalent diseases in terms of incidence and absolute case numbers. TB continues to dominate among adults—particularly farmers, homemakers, retirees, and commercial service workers—highlighting the need for ongoing screening, prompt case finding, and treatment adherence programs in rural areas. The high burden of influenza across all major groups (except farmers) underscores the importance of increasing influenza vaccination coverage, especially among school-aged children and high-contact occupational groups (e.g., commercial service workers). HFMD, varicella, and ID are common among children. In light of the Holm-adjusted pairwise results, ID and varicella are best interpreted as similar in terms of share at the county level and as closely trailing HFMD, which suggests joint prioritization within pediatric prevention and control efforts. At present, the coverage of the EV71 vaccine for HFMD is relatively low, and no comprehensive vaccination strategies for ID have been developed; namely, only imported oral rotavirus vaccines are available to prevent rotavirus diarrhea. Both vaccines are classified as Category II in this region and are thus characterized by lower levels of coverage than Category I vaccines (which might be referred to as a national immunization program in other countries or regions) [15–18]. Therefore, the incorporation of these relevant vaccines into the Category I immunization program would positively impact disease prevention and control in this context. Furthermore, owing to the limitations of vaccines [19, 20], the cultivation of healthy hygiene practices is also a crucial preventive measure, and the development of targeted health education programs may yield positive results [21–23]. 

Month-of-year seasonal indices (2013–2021) showed county-level peaks in July for TB, January for influenza, and June for HFMD, with peak-month concordance within ± 1 month across the six major groups of 2/6, 4/6, and 2/6, respectively (Supplementary Fig. S1). Thus, influenza exhibited moderate alignment between county and group peaks, whereas TB and HFMD showed partial alignment with dispersed group-specific peaks. This dispersion likely reflects heterogeneous exposure contexts and timing (e.g., school calendars and childcare settings for HFMD; work-related and age-related patterns for TB) and differences in care-seeking/reporting behavior. Operationally, the findings argue against a “one-size-fits-all” schedule for all groups, especially for TB and HFMD.

This study applied a classical SARIMA approach to predict three major statutory infectious diseases in Ziyang County—tuberculosis, influenza, and HFMD—thereby providing a quantitative basis for regional disease control planning. With respect to tuberculosis, the SARIMA forecasts aligned with those of traditional statistical analyses, confirming a sustained downward trend and clearly characterizing the seasonal peaks in spring and summer. These findings support the targeted timing of health education and screening programs. Influenza forecasts were complicated by structural breaks in surveillance and reporting. The use of sentinel operations was strengthened in 2017, and the 2020 diagnostic guidelines expanded the laboratory confirmation criteria, likely increasing case ascertainment. Our two-window sensitivity analysis (2013–2021 vs. 2018–2021) was designed to quantify the impact of these changes. Although the AIC values differed, short-horizon predictions were broadly similar, which is consistent with the property of the SARIMA model of downweighting distant history [7, 24, 25]. Taken together, earlier under-ascertainment (pre-2017) likely decreased historical levels but had a limited incremental influence on short-horizon forecasts once seasonal differencing and recent dynamics were incorporated. Nevertheless, the forecast errors for 2022–2023 highlight the effect of abrupt exogenous shocks—including the relaxation of COVID-19 control measures, behavioral rebound, and increased susceptibility—phenomena collectively referred to as “immunity debt” [26–28]. These shocks lie outside the scope of the univariate SARIMA model and partly explain the divergence between predicted and observed case counts. The prevalence of sentinel ILI is internationally recommended as a more stable indicator for modeling influenza trends [14]. However, county-level ILI denominators were unavailable; therefore, we modeled case counts and population-based incidence rates and interpreted these forecasts with caution. Future work should aim to incorporate ILI denominators or use state-space or structural-break models that explicitly account for surveillance changes. The SARIMA model performed poorly for HFMD, sometimes generating negative forecasts. The HFMD incidence is sporadic in this region, with many zero-count months, violating the assumption of stationarity and approximate normality of the SARIMA model [7, 24, 29]. Therefore, trend predictions based on the SARIMA model are limited in situations that involve sparse data or datasets featuring low case counts. In this study, HFMD was nevertheless included in the SARIMA analysis for consistency across the major reported infectious diseases and to explicitly demonstrate the boundaries of automated SARIMA modeling in real-world practice. This limitation underscores that the SARIMA model is best suited for diseases with relatively stable, seasonal incidence, whereas sparse or zero-inflated time series may require alternative models, such as zero-inflated Poisson or negative binomial regression, Bayesian hierarchical models, or hurdle-based time series approaches [30–32]. The seasonal windows recovered by the county-level SARIMA model are consistent in timing with the observed month-of-year profiles (Fig. 3; Supplementary Fig. S1), which supports a two-tier operational approach. At the county level, forecasts define preparedness windows (e.g., advance allocation of diagnostics, staffing, and communications). At the group level, the month-of-year profiles fine-tune timing and targeting: for influenza, school-based messaging and vaccination outreach can be brought forward by 2–4 weeks in student-dense settings; for TB, screening and follow-up can be staged in the 1–2 months preceding group-specific peaks among farmers, retirees, and homemakers; for HFMD, hand hygiene and environmental cleaning can be intensified earlier in childcare and lower-grade school environments. The occupational group rankings motivated our focus on three representative, high-burden diseases—TB (adult-dominated), HFMD (child/student-dominated), and influenza (cross-cutting). We did not extend the SARIMA model to subgroup-level series because several groups exhibited sparse or zero-inflated monthly counts, for which the univariate SARIMA model becomes unstable, as illustrated by HFMD at the county scale. Alternative approaches (e.g., zero-inflated Poisson/negative-binomial time series, hierarchical models, or state-space/cosinor variants) may better accommodate sparsity while borrowing strength across groups.

Seasonal indices summarize within-year proportions averaged across years and are intended for qualitative timing rather than formal inference. Years with zero totals contribute no information; in very sparse series, peak months and amplitudes may be unstable. The ± 1-month concordance threshold is heuristic; broader windows (e.g., ± 2 months) could be examined as a sensitivity analysis but were not examined here. Finally, indices for influenza were computed from case counts rather than from ILI denominators; incorporating denominators and exogenous drivers (e.g., influenza-like illness [ILI], meteorology, school calendars) within hierarchical or structural-break models represents an important direction for future work. Beyond these methodological considerations, following the adjustment of COVID-19 prevention and control policies, infectious disease control entered a new phase. As a result of the development of more convenient and accurate laboratory diagnostic methods, an increasing number of patients with notifiable infectious diseases can now be identified, reported, and distinguished from ordinary patients who exhibit only upper respiratory symptoms. Disease control agencies must continuously improve their technological methods and workflows. Interdisciplinary approaches to the tasks of infectious disease forecasting and intervention are coming to represent a future trend in efforts aimed at disease control [1, 33–35]. For example, reliable statistical and computational models can be employed to predict infectious disease trends automatically, issue early warnings regarding potential outbreaks, and implement targeted health education or intervention measures in advance [24, 35]. 

### Limitations

This study has several limitations. First, changes in influenza surveillance protocols and diagnostic practices likely influenced case ascertainment, which the SARIMA model may not fully capture. Second, the absence of ILI denominators limited our ability to normalize for fluctuations in care seeking and testing intensity. Finally, mid-year populations were estimated via linear interpolation between census years, which may not fully reflect short-term demographic changes. These factors should be considered when forecasts are interpreted.

## Conclusion

From 2013 to 2023, tuberculosis, influenza, and HFMD were the leading contributors to the burden of notifiable infectious diseases in Ziyang County. The incidence of tuberculosis declined steadily, whereas the incidence of influenza rebounded sharply in 2023 after nonpharmaceutical interventions were lifted. SARIMA models effectively captured seasonal patterns and generated short-term forecasts, although their accuracy was limited by surveillance updates and abrupt behavioral changes. Future work should incorporate sentinel ILI denominators and modeling approaches that account for structural breaks to enhance forecast reliability.

## Supplementary Information

Supplementary Material 1.

## Data Availability

The datasets and Python scripts used in this study are available from the corresponding author upon reasonable request and may be provided to qualified researchers for the purpose of reproducing the results.

## Abbreviations

AIC: Akaike information criterion
AR: Autoregressive
ARIMA: Autoregressive integrated moving average
CDC (CDC(s)): Centers for Disease Control and Prevention
COVID-19: Coronavirus disease 2019
HFMD: Hand–foot–mouth disease
ID: Infectious diarrheal disease
ILI: Influenza-like illness
MA: Moving average
MAE: Mean absolute error
MAPE: Mean absolute percentage error
NAAT(s): Nucleic acid amplification test(s)
PI: Prediction interval
RMSE: Root mean squared error
R^2^: Coefficient of determination
SARIMA: Seasonal autoregressive integrated moving average
WHO: World Health Organization
