# Supplementary material for: Trends in epidemics pertaining to notifiable infectious diseases in China and prediction models for key diseases: a case study of Ziyang County
Source: BMC Public Health. 2025 Nov 21;25:4099. doi: 10.1186/s12889-025-25482-2 (PMC12639878; doi:10.1186/s12889-025-25482-2)
Supplement: Supplementary file 1 — Supplementary Material 1. [file 12889_2025_25482_MOESM1_ESM.docx]

**Supplementary Materials**

Supplementary Table S1. Annual number of reported cases and incidence rates (per 10,000 population) of notifiable infectious diseases in Ziyang County, 2013–2023.

Supplementary Table S1a.

Annual reported cases and incidence rates (per 10,000 population) of tuberculosis (TB), influenza, hand–foot–mouth disease (HFMD), other infectious diarrheal disease (ID), varicella, and hepatitis B in Ziyang County, 2013–2023.

| Year | TB N  (Rate) | Influenza N  (Rate) | HFMD N  (Rate) | ID N  (Rate) | Varicella N  (Rate) | Hepatitis B N  (Rate) |
| --- | --- | --- | --- | --- | --- | --- |
| 2013 | 610 (20.4) | 9 (0.3) | 355 (11.9) | 248 (8.3) | 337 (11.3) | 161 (5.4) |
| 2014 | 604 (19.8) | 11 (0.4) | 128 (4.2) | 251 (8.2) | 256 (8.4) | 239 (7.8) |
| 2015 | 687 (22.1) | 7 (0.2) | 371 (11.9) | 165 (5.3) | 244 (7.9) | 192 (6.2) |
| 2016 | 646 (20.4) | 6 (0.2) | 388 (12.2) | 336 (10.6) | 265 (8.4) | 205 (6.5) |
| 2017 | 731 (22.6) | 4 (0.1) | 379 (11.7) | 388 (12.0) | 261 (8.1) | 223 (6.9) |
| 2018 | 573 (17.4) | 64 (1.9) | 376 (11.4) | 408 (12.4) | 330 (10.0) | 134 (4.1) |
| 2019 | 538 (17.3) | 450 (14.5) | 616 (19.8) | 356 (11.5) | 429 (13.8) | 129 (4.2) |
| 2020 | 449 (15.4) | 514 (17.6) | 523 (17.9) | 430 (14.7) | 477 (16.3) | 141 (4.8) |
| 2021 | 445 (16.3) | 322 (11.8) | 258 (9.4) | 469 (17.2) | 232 (8.5) | 145 (5.3) |
| 2022 | 384 (15.1) | 1000 (39.3) | 213 (8.4) | 232 (9.1) | 224 (8.8) | 168 (6.6) |
| 2023 | 437 (18.5) | 2140 (90.8) | 464 (19.7) | 206 (8.7) | 185 (7.9) | 155 (6.6) |
| Total | 6104 (18.8) | 4527 (13.9) | 4071 (12.5) | 3489 (10.7) | 3240 (10.0) | 1892 (5.83) |

N: Number of reported cases; Rate: cases per 10,000 people.

Supplementary Table S1b.

Annual reported cases and incidence rates (per 10,000 population) of mumps, syphilis, hepatitis C, COVID-19, acute hemorrhagic conjunctivitis (AHC), and HIV/AIDS in Ziyang County, 2013–2023.

| Year | Mumps N  (Rate) | Syphilis N  (Rate) | Hepatitis C N  (Rate) | COVID-19 N  (Rate) | AHC N  (Rate) | HIV/AIDS N  (Rate) |
| --- | --- | --- | --- | --- | --- | --- |
| 2013 | 514 (17.2) | 100 (3.4) | 22 (0.7) | 0 (0.0) | 56 (1.9) | 14 (0.5) |
| 2014 | 197 (6.5) | 112 (3.7) | 33 (1.1) | 0 (0.0) | 40 (1.3) | 4 (0.1) |
| 2015 | 67 (2.2) | 111 (3.6) | 26 (0.8) | 0 (0.0) | 50 (1.6) | 7 (0.2) |
| 2016 | 66 (2.1) | 75 (2.4) | 41 (1.3) | 0 (0.0) | 19 (0.6) | 19 (0.6) |
| 2017 | 31 (1.0) | 109 (3.4) | 53 (1.6) | 0 (0.0) | 1 (0.0) | 10 (0.3) |
| 2018 | 46 (1.4) | 91 (2.8) | 66 (2.0) | 0 (0.0) | 0 (0.0) | 21 (0.6) |
| 2019 | 42 (1.4) | 87 (2.8) | 72 (2.3) | 0 (0.0) | 0 (0.0) | 15 (0.5) |
| 2020 | 29 (1.0) | 77 (2.6) | 43 (1.5) | 15 (0.5) | 5 (0.2) | 12 (0.4) |
| 2021 | 42 (1.5) | 74 (2.7) | 25 (0.9) | 1 (0.0) | 0 (0.0) | 9 (0.3) |
| 2022 | 30 (1.2) | 39 (1.5) | 23 (0.9) | 116 (4.6) | 1 (0.0) | 16 (0.6) |
| 2023 | 16 (0.7) | 56 (2.4) | 11 (0.5) | 219 (9.3) | 6 (0.3) | 7 (0.3) |
| Total | 1080 (3.3) | 931 (2.9) | 415 (1.3) | 351 (1.1) | 178 (0.5) | 134 (0.4) |

Supplementary Table S1c.

Annual reported cases and incidence rates (per 10,000 population) of measles, bacillary dysentery (BD), gonorrhea, Japanese encephalitis (JE), hepatitis E, and hepatitis A in Ziyang County, 2013–2023.

| Year | Measles N  (Rate) | BD N  (Rate) | Gonorrhea N  (Rate) | JE N  (Rate) | Hepatitis E N  (Rate) | Hepatitis A N  (Rate) |
| --- | --- | --- | --- | --- | --- | --- |
| 2013 | 2 (0.1) | 14 (0.5) | 0 (0.0) | 14 (0.5) | 7 (0.2) | 5 (0.2) |
| 2014 | 3 (0.1) | 12 (0.4) | 1 (0.0) | 5 (0.2) | 3 (0.1) | 1 (0.0) |
| 2015 | 58 (1.9) | 9 (0.3) | 3 (0.1) | 7 (0.2) | 1 (0.0) | 0 (0.0) |
| 2016 | 6 (0.2) | 6 (0.2) | 4 (0.1) | 2 (0.1) | 1 (0.0) | 3 (0.1) |
| 2017 | 4 (0.1) | 3 (0.1) | 6 (0.2) | 4 (0.1) | 3 (0.1) | 5 (0.2) |
| 2018 | 2 (0.1) | 1 (0.0) | 5 (0.2) | 1 (0.0) | 3 (0.1) | 1 (0.0) |
| 2019 | 2 (0.1) | 2 (0.1) | 7 (0.2) | 1 (0.0) | 6 (0.2) | 6 (0.2) |
| 2020 | 1 (0.0) | 0 (0.0) | 7 (0.2) | 1 (0.0) | 0 (0.0) | 0 (0.0) |
| 2021 | 0 (0.0) | 0 (0.0) | 5 (0.2) | 2 (0.1) | 5 (0.2) | 3 (0.1) |
| 2022 | 6 (0.2) | 1 (0.0) | 3 (0.1) | 2 (0.1) | 2 (0.1) | 1 (0.0) |
| 2023 | 1 (0.0) | 1 (0.0) | 5 (0.2) | 0 (0.0) | 7 (0.3) | 0 (0.0) |
| Total | 85 (0.3) | 49 (0.2) | 46 (0.1) | 39 (0.1) | 38 (0.1) | 25 (0.1) |

Supplementary Table S1d.

Annual reported cases and incidence rates (per 10,000 population) of pertussis, brucellosis, scarlet fever, rubella, rabies, and malaria in Ziyang County, 2013–2023.

| Year | Pertussis N  (Rate) | Brucellosis N  (Rate) | Scarlet fever N  (Rate) | Rubella N  (Rate) | Rabies N  (Rate) | Malaria N  (Rate) |
| --- | --- | --- | --- | --- | --- | --- |
| 2013 | 0 (0.0) | 0 (0.0) | 0 (0.0) | 1 (0.0) | 0 (0.0) | 0 (0.0) |
| 2014 | 0 (0.0) | 0 (0.0) | 0 (0.0) | 2 (0.1) | 1 (0.0) | 1 (0.0) |
| 2015 | 1 (0.0) | 4 (0.1) | 2 (0.1) | 2 (0.1) | 0 (0.0) | 0 (0.0) |
| 2016 | 1 (0.0) | 1 (0.0) | 1 (0.0) | 1 (0.0) | 0 (0.0) | 0 (0.0) |
| 2017 | 0 (0.0) | 1 (0.0) | 2 (0.1) | 0 (0.0) | 2 (0.1) | 0 (0.0) |
| 2018 | 1 (0.0) | 1 (0.0) | 3 (0.1) | 0 (0.0) | 2 (0.1) | 1 (0.0) |
| 2019 | 5 (0.2) | 0 (0.0) | 1 (0.0) | 0 (0.0) | 0 (0.0) | 1 (0.0) |
| 2020 | 2 (0.1) | 1 (0.0) | 1 (0.0) | 0 (0.0) | 0 (0.0) | 0 (0.0) |
| 2021 | 0 (0.0) | 1 (0.0) | 0 (0.0) | 0 (0.0) | 0 (0.0) | 0 (0.0) |
| 2022 | 4 (0.2) | 1 (0.0) | 0 (0.0) | 1 (0.0) | 0 (0.0) | 0 (0.0) |
| 2023 | 4 (0.2) | 0 (0.0) | 0 (0.0) | 0 (0.0) | 0 (0.0) | 0 (0.0) |
| Total | 18 (0.1) | 10 (0.0) | 10 (0.0) | 7 (0.0) | 5 (0.0) | 3 (0.0) |

Supplementary Table S1e.

Annual reported cases and incidence rates (per 10,000 population) of typhoid fever, hemorrhagic fever with renal syndrome (HFRS), kala-azar, dengue fever, and leprosy in Ziyang County, 2013–2023.

| Year | Typhoid fever N  (Rate) | HFRS N  (Rate) | Kala-azar N  (Rate) | Dengue fever N  (Rate) | Leprosy N  (Rate) |
| --- | --- | --- | --- | --- | --- |
| 2013 | 0 (0.0) | 0 (0.0) | 0 (0.0) | 0 (0.0) | 1 (0.0) |
| 2014 | 0 (0.0) | 0 (0.0) | 0 (0.0) | 0 (0.0) | 0 (0.0) |
| 2015 | 0 (0.0) | 1 (0.0) | 0 (0.0) | 0 (0.0) | 0 (0.0) |
| 2016 | 1 (0.0) | 0 (0.0) | 0 (0.0) | 0 (0.0) | 0 (0.0) |
| 2017 | 0 (0.0) | 0 (0.0) | 0 (0.0) | 0 (0.0) | 0 (0.0) |
| 2018 | 0 (0.0) | 1 (0.0) | 0 (0.0) | 0 (0.0) | 0 (0.0) |
| 2019 | 1 (0.0) | 0 (0.0) | 0 (0.0) | 1 (0.0) | 0 (0.0) |
| 2020 | 1 (0.0) | 0 (0.0) | 1 (0.0) | 0 (0.0) | 0 (0.0) |
| 2021 | 0 (0.0) | 0 (0.0) | 1 (0.0) | 0 (0.0) | 0 (0.0) |
| 2022 | 0 (0.0) | 0 (0.0) | 0 (0.0) | 0 (0.0) | 0 (0.0) |
| 2023 | 0 (0.0) | 0 (0.0) | 0 (0.0) | 0 (0.0) | 0 (0.0) |
| Total | 3 (0.0) | 2 (0.0) | 2 (0.0) | 1 (0.0) | 1 (0.0) |

Supplementary Table S1f.

Compared with adjacent pairs (Holm-adjusted)

| Rank | Pair | Holm-adjusted p | Sig. |
| --- | --- | --- | --- |
| 1 | TB – Influenza | 1.17e-50 | *** |
| 2 | Influenza – HFMD | 8.83e-05 | *** |
| 3 | HFMD – ID | 2.88e-09 | *** |
| 4 | ID – Varicella | 0.17 | ns |
| 5 | Varicella – Hepatitis B | 1.76e-77 | *** |
| 6 | Hepatitis B – Mumps | 2.10e-48 | *** |
| 7 | Mumps –Syphilis | 0.07 | ns |
| 8 | Syphilis –Hepatitis C | 1.26e-43 | *** |
| 9 | Hepatitis C – COVID-19 | 1.00 | ns |
| 10 | COVID-19 – AHC | 6.26e-12 | *** |
| 11 | AHC – HIV/AIDS | 0.86 | ns |
| 12 | HIV/AIDS – Measles | 0.08 | ns |
| 13 | Measles – BD | 0.16 | ns |
| 14 | BD – Gonorrhea | 1.00 | ns |
| 15 | Gonorrhea – JE | 1.00 | ns |
| 16 | JE – Hepatitis E | 1.00 | ns |
| 17 | Hepatitis E – Hepatitis A | 1.00 | ns |
| 18 | Hepatitis A – Pertussis | 1.00 | ns |
| 19 | Pertussis – Brucellosis | 1.00 | ns |
| 20 | Brucellosis –Scarlet fever | 1.00 | ns |
| 21 | Scarlet fever – Rubella | 1.00 | ns |
| 22 | Rubella – Rabies | 1.00 | ns |
| 23 | Rabies – Malaria | 1.00 | ns |
| 24 | Malaria – Typhoid fever | 1.00 | ns |
| 25 | Typhoid fever – HFRS | 1.00 | ns |
| 26 | HFRS – Kala-azar | 1.00 | ns |
| 27 | Kala-azar – Dengue fever | 1.00 | ns |
| 28 | Dengue fever – Leprosy | 1.00 | ns |

*** p<0.001; ** p<0.01; * p<0.05; ns ≥0.05

Overall differences were assessed by Pearson chi-square goodness-of-fit (null: equal proportions). Pairwise differences were tested on the two-category margin (two-sided), with Holm-adjusted *p* values controlling the familywise error rate.

Supplementary Table S2.

Annual number of reported cases of notifiable infectious diseases by population group, Ziyang County, 2013–2023.

| Rank | Population Group | Cases | Proportion (%) |
| --- | --- | --- | --- |
| 1 | Children | 10,465 | 39.11% |
| 2 | Farmers | 8,690 | 32.48% |
| 3 | Students | 5,285 | 19.75% |
| 4 | Homemakers | 827 | 3.09% |
| 5 | Retirees | 244 | 0.91% |
| 6 | Commercial Services | 213 | 0.80% |
| 7 | Unknown | 209 | 0.78% |
| 8 | Workers | 181 | 0.68% |
| 9 | Government Staff | 174 | 0.65% |
| 10 | Migrant Workers | 153 | 0.57% |
| 11 | Others | 107 | 0.40% |
| 12 | Teachers | 104 | 0.39% |
| 13 | Medical Personnel | 54 | 0.20% |
| 14 | Catering & Food | 23 | 0.09% |
| 15 | Public Venue Staff | 14 | 0.05% |
| 16 | Herdsmen | 9 | 0.03% |
| 17 | Childcare/Housekeepers | 2 | 0.01% |
| 18 | Seamen/Drivers | 1 | 0.00% |
| 19 | Fishermen | 1 | 0.00% |

Unknown denotes records lacking occupation information on case report cards.

Supplementary Table S3.

AIC comparison of the candidate SARIMA models for the four major diseases.

| Disease | Year Range | Selected | SARIMA Model Specification | Intercept Included | AIC |
| --- | --- | --- | --- | --- | --- |
| TB | 2013–2021 | Yes | (2,1,1)(2,1,0)[12] | No | 787.224 |
| TB | 2013–2021 | No | (2,1,1)(1,1,0)[12] | No | 787.979 |
| TB | 2013–2021 | No | (3,1,1)(2,1,0)[12] | No | 788.63 |
| TB | 2013–2021 | No | (2,1,2)(2,1,0)[12] | No | 788.845 |
| TB | 2013–2021 | No | (1,1,1)(2,1,0)[12] | No | 789.307 |
| HFMD | 2013–2021 | Yes | (0,1,0)(2,1,1)[12] | No | 988.171 |
| HFMD | 2013–2021 | No | (0,1,0)(1,1,2)[12] | No | 988.472 |
| HFMD | 2013–2021 | No | (0,1,1)(2,1,1)[12] | No | 989.236 |
| HFMD | 2013–2021 | No | (1,1,0)(2,1,1)[12] | No | 989.929 |
| HFMD | 2013–2021 | No | (0,1,0)(2,1,2)[12] | No | 990.088 |
| Influenza | 2013–2021 | Yes | (0,1,1)(1,1,1)[12] | No | 989.948 |
| Influenza | 2013–2021 | No | (0,1,1)(2,1,0)[12] | No | 990.066 |
| Influenza | 2013–2021 | No | (0,1,1)(0,1,2)[12] | No | 991.761 |
| Influenza | 2013–2021 | No | (0,1,1)(1,1,1)[12] | Yes | 991.887 |
| Influenza | 2013–2021 | No | (0,1,1)(2,1,1)[12] | No | 991.901 |
| Influenza | 2018–2021 | Yes | (0,1,0)(1,1,0)[12] | No | 402.847 |
| Influenza | 2018–2021 | No | (0,1,1)(1,1,0)[12] | No | 403.83 |
| Influenza | 2018–2021 | No | (0,1,0)(1,1,0)[12] | Yes | 404.418 |
| Influenza | 2018–2021 | No | (1,1,0)(1,1,0)[12] | No | 404.647 |
| Influenza | 2018–2021 | No | (0,1,0)(0,1,0)[12] | No | 421.561 |

Supplementary Fig. S1


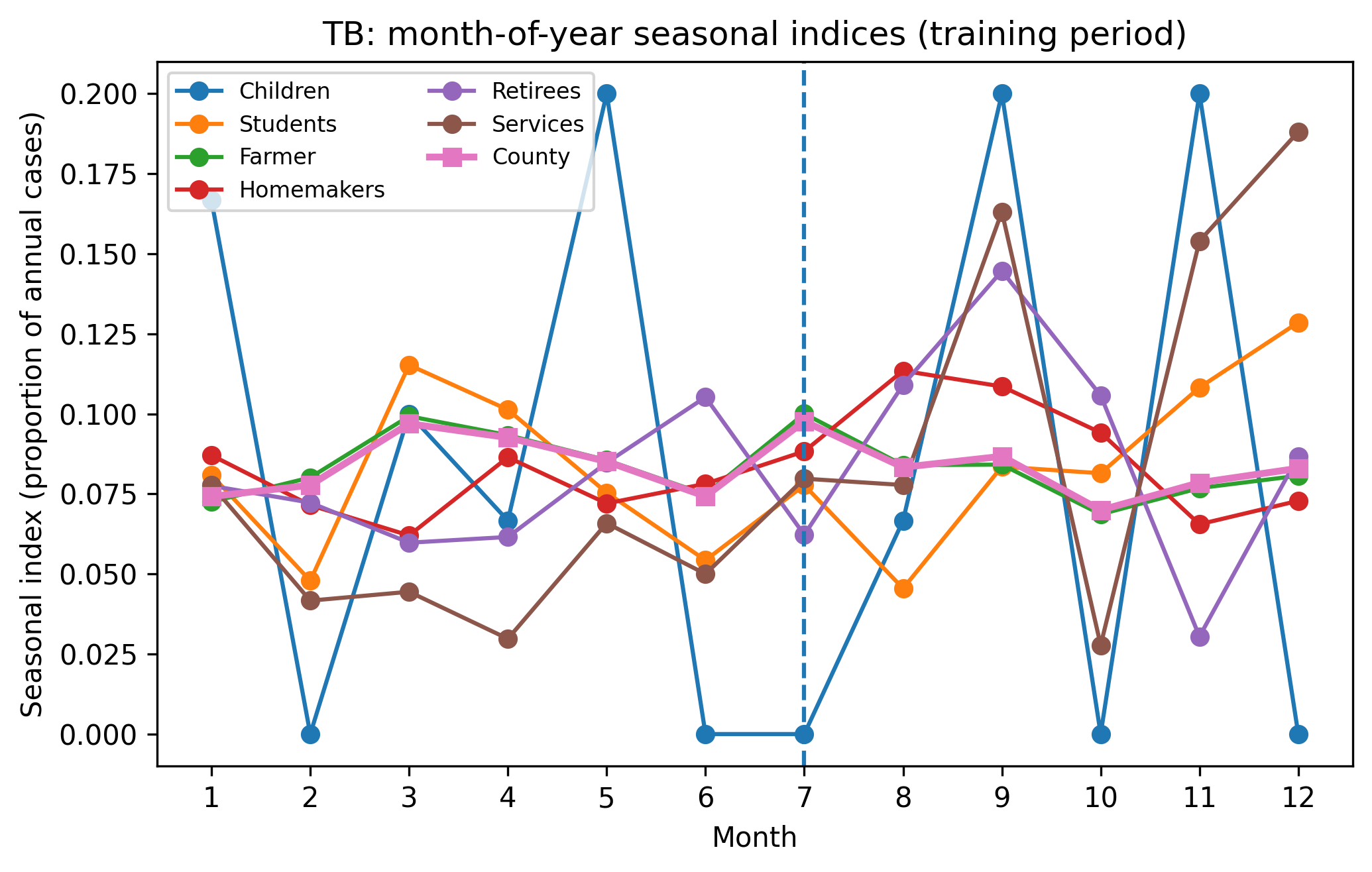


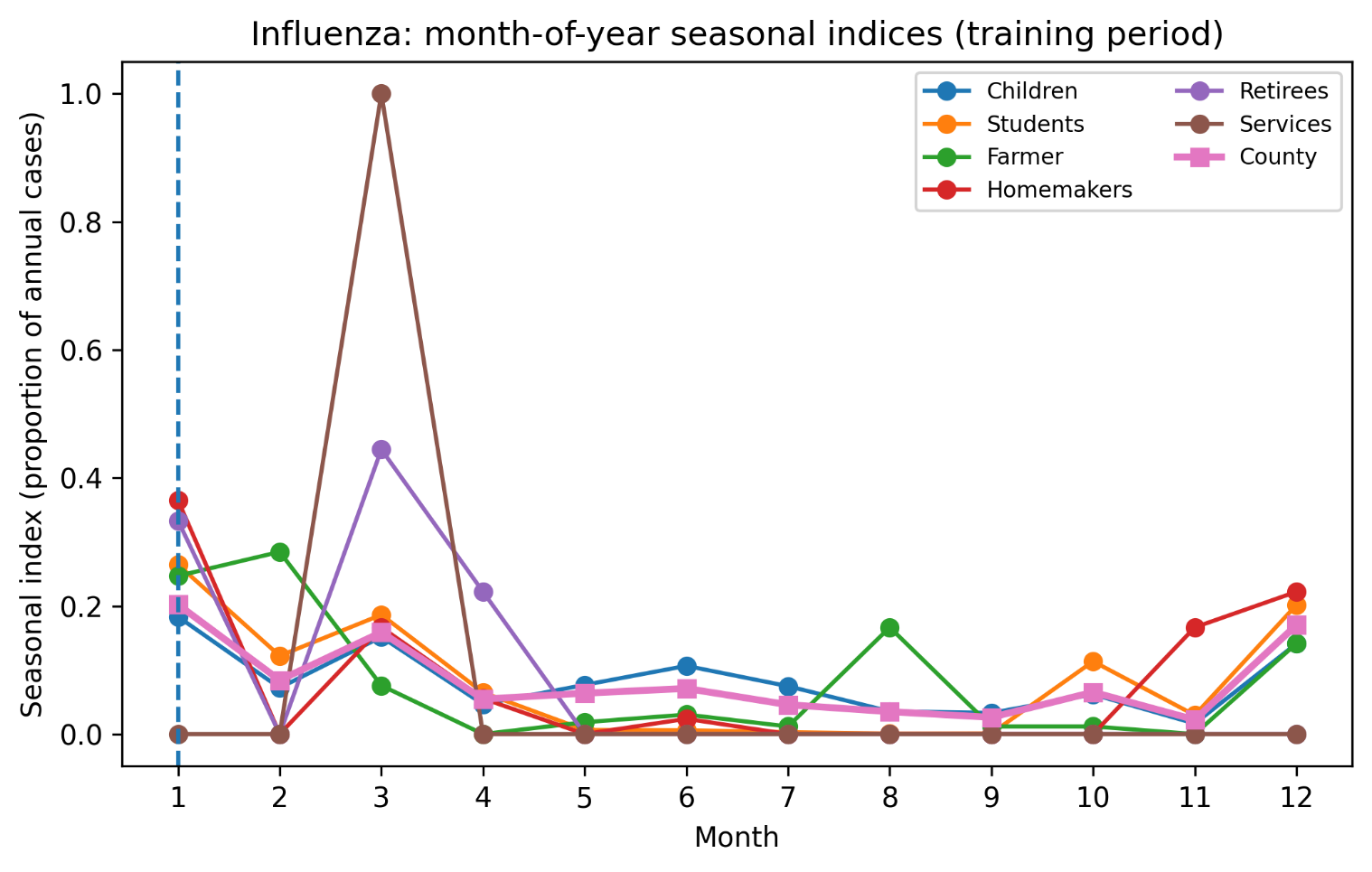


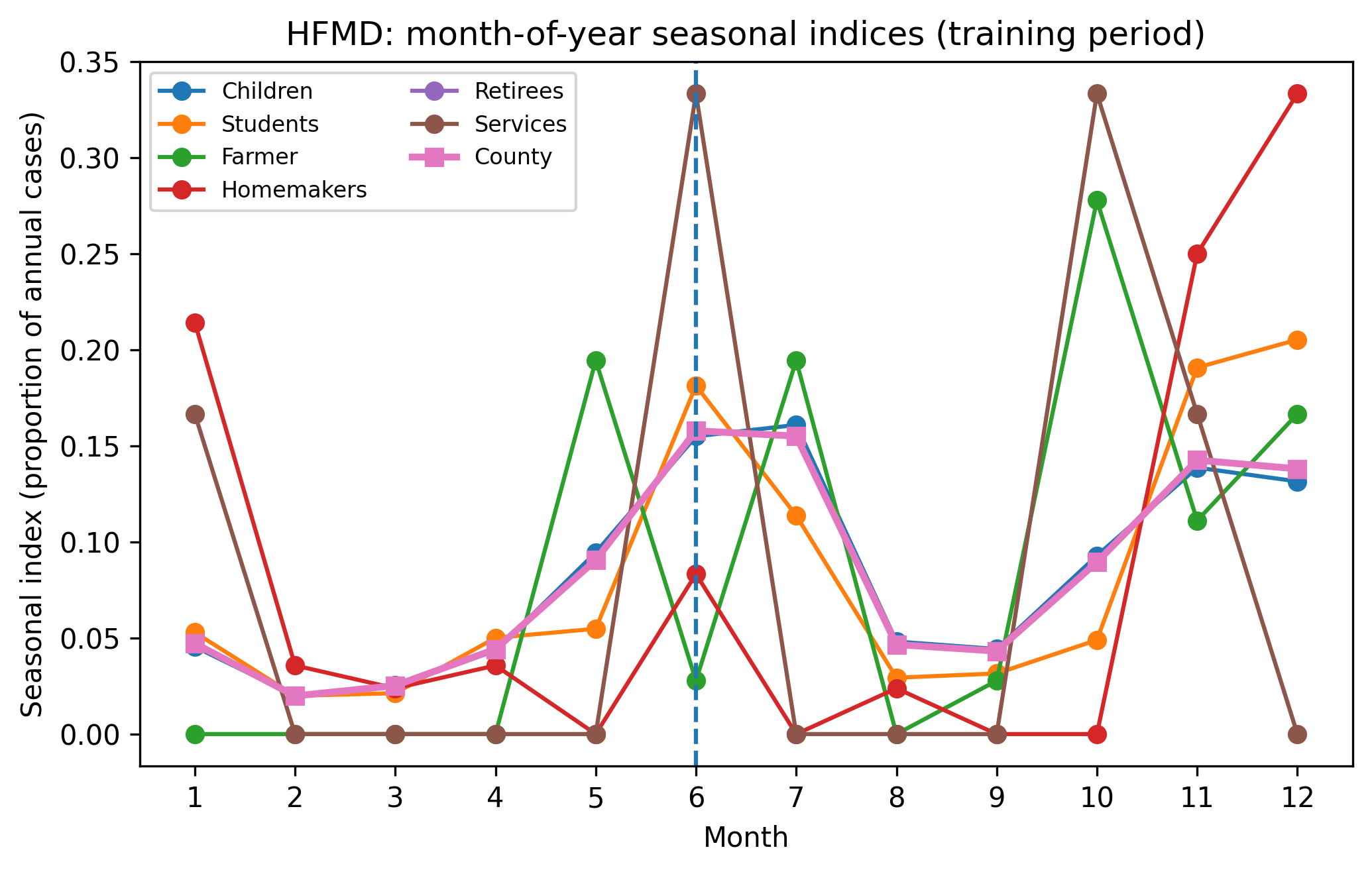


Month-of-year seasonal indices (2013–2021) for (A) TB, (B) influenza, and (C) HFMD at the county and group levels. Lines show group-specific indices; the thicker line shows the county curve. The vertical dashed line indicates the county peak month. The peak-month concordances (±1 month) were 2/6 (TB), 4/6 (influenza), and 2/6 (HFMD).
